# *BRCA1* and *BRCA2* Mutations in Polish Women with Ductal Carcinoma In Situ

**DOI:** 10.3390/cancers17040613

**Published:** 2025-02-11

**Authors:** Sylwia Feszak, Igor Jarosław Feszak, Wojciech Kluźniak, Dominika Wokołorczyk, Klaudia Stempa, Katarzyna Gliniewicz, Jan Uciński, Tomasz Huzarski, Tadeusz Dębniak, Jacek Gronwald, Jan Lubiński, Steven A. Narod, Cezary Cybulski

**Affiliations:** 1 International Hereditary Cancer Center, Department of Genetics and Pathology, Pomeranian Medical University in Szczecin, 71-252 Szczecin, Poland; igorfeszak@gmail.com (I.J.F.); wojciech.kluzniak@pum.edu.pl (W.K.); dominikawok@o2.pl (D.W.); klaudia.stempa@pum.edu.pl (K.S.); katarzyna.gliniewicz@pum.edu.pl (K.G.); jan.ucinski@pum.edu.pl (J.U.); tomasz.huzarski@pum.edu.pl (T.H.); tadeusz.debniak@pum.edu.pl (T.D.); jacek.gronwald@pum.edu.pl (J.G.); jan.lubinski@pum.edu.pl (J.L.); 2 Department of Pediatrics, Pediatric Oncology, and Immunology, Pomeranian Medical University, 71-252 Szczecin, Poland; 3 Institute of Health Sciences, Pomeranian University in Słupsk, 76-200 Słupsk, Poland; 4 Department of Clinical Genetics and Pathology, University of Zielona Góra, 65-046 Zielona Góra, Poland; 5 Women’s College Research Institute, Women’s College Hospital, Toronto, ON M5S 1B2, Canada; steven.narod@wchospital.ca; 6 Dalla Lana School of Public Health, University of Toronto, Toronto, ON M5T 3M7, Canada

**Keywords:** ductal carcinoma in situ, Polish population, *BRCA1* gene, *BRCA2* gene, germline mutations

## Abstract

**Background/Objectives**: Ductal carcinoma in situ (DCIS) is the most common non-invasive form of breast cancer. It is not clear to what extent DCIS is a part of the hereditary breast/ovarian cancer syndrome caused by *BRCA1/2* mutations. Therefore, we investigated the association of *BRCA1/2* mutations in patients with DCIS and assessed their impact on survival. **Methods**: We studied 564 Polish women with DCIS for six alleles in *BRCA1* (c.181T>G, c.5266dupC, c.4035delA, c.3700_3704del5, c.68_69del and c.5251C>T) and four in *BRCA2* (c.658_659del, c.3847_3848del, c.5946del and c.7913_7917del). To investigate the association of *BRCA1/2* founder mutations with DCIS risk, we tested 4702 controls as a reference. To analyze survival, mutation carriers were followed for an average of 110 months. **Results**: A *BRCA1* mutation was present in seven (1.24%) cases and in twenty-two (0.47%) controls (OR = 3.27, 95%CI 1.36 to 7.87, *p* = 0.01). A *BRCA2* mutation was present in eight (1.42%) cases versus six (0.13%) controls (OR = 11.3, 95%CI 3.9 to 32.6, *p* < 0.0001). Three of the fifteen cases with *BRCA1/2* mutations developed invasive ipsilateral or contralateral breast cancer, on average 6 years from the diagnosis of DCIS. There were no deaths reported among the 15 mutation carriers with DCIS. **Conclusions**: DCIS is a part of the hereditary breast/ovarian cancer syndrome caused by *BRCA1/2* mutations. Women with DCIS should receive genetic counseling and testing for *BRCA1/2* mutations. *BRCA1/2* mutations may predispose women to a better DCIS prognosis, but further studies are needed.

## Simple Summary

Ductal carcinoma in situ (DCIS) is the most common form of non-invasive breast cancer. The aim of our study was to investigate the role of *BRCA1/2* mutations in the etiology of DCIS and their impact on survival among patients with DCIS. We screened 564 Polish women with DCIS for ten Polish common founder mutations in *BRCA1/2* (six in *BRCA1*, four in *BRCA2*). A mutation was detected in fifteen cases (2.7%), including seven in *BRCA1* and eight in *BRCA2*. A case-control study revealed the association of both *BRCA2* (OR = 11.3, 95%CI 3.9 to 32.6, *p* < 0.0001) and *BRCA1* (OR = 3.27, 95%CI 1.36 to 7.87, *p* = 0.01) with DCIS. Across the follow-up period (mean: 110 months), there were no deaths reported among the 15 mutation carriers with DCIS. We conclude that women with DCIS should receive genetic counseling and testing for *BRCA1/2* mutations. *BRCA1/2* mutations may predispose women to a better DCIS prognosis, but further studies are needed.

## 1. Introduction

Breast cancer is the most frequent malignant tumor in women, annually diagnosed in over two million females worldwide. The lifetime risk of breast cancer (BC) is approximately 8–15% across different ethnic groups, including approximately 8% in Poland [1,2]. Most cases of breast cancer (90%) are invasive and are an important cause of morbidity and mortality from cancer. Only 10–15% of breast cancer cases are non-invasive tumors, which are usually associated with a good prognosis [3].

Ductal carcinoma in situ (DCIS) is the most common form of non-invasive breast cancer. It is responsible for about 80–90% of all non-invasive tumors of the breast. Most cases of DCIS (90%) are asymptomatic and are usually detected through screening mammography. However, 10% of DCIS tumors are symptomatic, i.e., they cause nipple discharge, Paget’s disease of the nipple or are diagnosed as a palpable tumor mass [4]. The common implementation of screening mammography in recent years has led to a three-fold increase in the diagnosis of preinvasive breast cancer in the UK, but only a 24% increase in the diagnosis rate of invasive breast cancer (IBC) [3].

DCIS is considered a non-obligate precursor of invasive breast cancer. However, the natural history of DCIS is not completely understood. It is also unclear to what extent genetic predisposition plays a role in this process [5]. According to Petridis et al., there is a common genetic background between DCIS and invasive ductal carcinoma of the breast. They found that the majority of SNPs associated with invasive breast cancer are also associated with a predisposition to DCIS [6]. Barclay et al. conducted a cross-sectional study of 40,000 women and showed that risk factors such as overweightedness, nulliparous, late motherhood and a family history of breast cancer were similar for DCIS and invasive breast cancer [7]. Peila et al. conducted a prospective study of 273,000 women and found obesity among post-menopausal women was associated with increased risk of DCIS and IBC [8]. Other studies also reported similarities in environmental risk factors for DCIS and IBC [9,10]. In the Million Women Study, the magnitude of association with a positive family history of breast cancer in first or second-degree relatives was similar for women with DCIS and invasive cancer [11].

Approximately 10–15% of all breast cancer cases are hereditary, but the percentage depends on the ethnic group under study. The proportion is also higher in patients with a family history of breast or ovarian cancer. Deleterious mutations in the *BRCA1* and *BRCA2* genes are the principal cause of hereditary breast and ovarian cancer syndrome (HBOC) [12,13]. *BRCA1/2* mutation carriers with a family history of breast cancer were found to have a higher risk of breast cancer than those who report no breast cancer in their relatives [14]. DCIS is increasingly accepted to be a component of HBOC syndrome [15,16]. Previous studies from the UK, USA, Canada and Japan have reported a higher frequency of *BRCA1/2* mutations in women with DCIS compared to healthy subjects [17,18,19,20,21]. However, the natural history of DCIS among mutation carriers is unclear, i.e., whether the progression rate to invasive cancer is higher or if the DCIS is more often lethal in carriers than in non-carriers. It is thought that the preinvasive phase might be shorter among *BRCA1/2* mutation carriers than non-carriers, which makes DCIS more difficult to recognize [22]. Further, the optimal treatment and surveillance protocol for women with DCIS and a *BRCA1/2* mutation has not been determined.

Poland is a homogenous country from a genetic perspective. In Poland, the three most common founder mutations of *BRCA1* (c.181T>G, c.4035del and c.5266dup) were identified in 2000 [23,24,25]. Since then, additional recurrent mutations have been detected in the Polish population, and now a panel of 10–12 *BRCA1/2* variants is commonly used as an initial screening tool for breast and ovarian cancer risk assessments in the population. This testing panel includes the c.181T>G, c.4035del, c.5266dup, c.3700_3704del, c.68_69del and c.5251C>T mutations of BRCA1, and the c.658_659del, c.3847_3848del, c.5946del and c.7913_7917del variants of BRCA2. These founder alleles account for over 80% of all pathogenic mutations in *BRCA1* and *BRCA2* genes detected in a series of 1018 Polish families with hereditary breast and/or ovarian cancer [26].

The objectives of this study were to establish the frequency of the 10 common Polish founder mutations of *BRCA1/2* among Polish women with DCIS, to investigate whether or not these mutations confer an increased risk of DCIS in the Polish population, and to investigate the clinical characteristics and survival of *BRCA1/2* mutation carriers with DCIS.

## 2. Materials and Methods

### 2.1. Patients

We studied a group of women diagnosed with DCIS between 1997 and 2019, unselected for age and family history. We selected these patients from a registry of 17,086 unselected breast cancer cases housed at the Hereditary Cancer Center in Szczecin. Women with a history of invasive breast cancer (IBC), intralobular cancer or ovarian cancer prior to the diagnosis of DCIS were excluded.

A total of 1238 women with DCIS were identified in the electronic database, and clinical data confirming DCIS with or without micro-invasion and a pedigree were available for 830 cases. A high quality DNA sample was available for 586 women. We then restricted the study group to those for whom blood samples for DNA isolation were collected within 2 years of the date of diagnosis. Finally, 564 women were included in the current study. All enrolled patients signed a written informed consent form for genetic testing of their DNA samples at the time of blood draw.

All women were of Polish ethnicity and most (85%) were residents of north-western Poland. Each participant was either self- or physician-referred for genetic counseling. The diagnosis of DCIS was confirmed by a pathology report in all cases. Clinical, demographic and self-reported ancestry information was obtained at the time of the interview and the blood draw for DNA isolation. All cancer cases in first and second-degree relatives and their ages of onset were recorded.

Survival data (vital status: alive or dead; and if deceased, the date of death, including day, month and year) were requested from the Polish Ministry of the Interior and Administration in January 2020, and were obtained in February 2020. We obtained survival data for all patients. This study was approved by the Ethics Committee of Pomeranian Medical University in Szczecin (IRB No. KB-0012/34/05/2020/Z).

The mean age of diagnosis of DCIS among the 564 women was 55.3 years (median 55.0, range 23–91 years, SD 10.8); 27.3% (154/564) of women were diagnosed with DCIS before the age of 50 and 7.1% (40/564) of women were diagnosed before the age of 40. In 145 (25.7%) cases, there was a positive family history of breast cancer in one or more first or second-degree relative. Thirty (5.3%) women with DCIS reported a positive family history of ovarian cancer in at least one first or second-degree relative. Among the cases with a positive family history, 40 women were from families with 3 or more affected women with breast or ovarian cancer (7.1% of all 564 patients with DCIS).

For women who tested positive for a mutation in the *BRCA1* or *BRCA2* genes, we collected detailed clinical data using a standardized follow-up questionnaire, including pathology, the presence of microinvasion, ER, PR and HER2 status, the means of treatment, and the diagnosis of invasive breast cancer after DCIS during the mean follow-up period of 110 months.

### 2.2. Controls

The purpose of the control group was to estimate the frequency of *BRCA1* and *BRCA2* founder alleles in the Polish population. The control group was recruited from four sources and included 4702 cancer-free women aged 20 to 94 years (mean, 53.0; median, 54.0; SD, 11.5) [27]. The first group consisted of 959 female residents of the Szczecin area matched by age (range 24 to 84 years) and place of residence to a series of patients with incident IBC diagnosed in Szczecin between 1996 and 2004, and were interviewed in 2007. The second subgroup consisted of 1717 unselected women (aged 32 to 72 years) who donated a blood sample for DNA analysis while undergoing breast ultrasound or mammography between 2009 and 2011 at nine different centers across Poland (Kielce, Kraków, Legnica, Olsztyn, Poznań, Szczecin, Świdnica, Toruń and Zielona Góra). Women with IBC and women with a positive family history of IBC were excluded from the control group. The third control group consisted of 1036 unselected women (20 to 94 years) randomly selected from computerized lists of primary care physicians in the Opole area and were invited to participate by e-mail in 2012–2013. The fourth group was recruited in 2007–2010 in Białystok, Łódź and Szczecin, from participants enrolled in a population colonoscopy screening program for colorectal cancer, and included 990 Polish women (50 to 66 years), who donated blood samples for DNA analysis. Only individuals of Polish ethnicity were enrolled in the case and control groups (all cases and controls were ethnic Poles).

### 2.3. Genotyping

*Cases*

We genotyped our individuals with DCIS for the six most common Polish founder mutations of *BRCA1* including c.5266dup (p.Gln1756Profs), c.4035del (p.Glu1346Lysfs), c.181 T > G (p.Cys61Gly), c.3700_3704del (p.Val1234Glnfs), c.68_69del (p.Glu23Valfs) and c.5251C > T (p.Arg1751Ter); and four recurrent mutations of *BRCA2*: c.658_659delGT (p.Val220Ilefs), c.3847_3848del (p.Val1283fs), c.5946del (p.Ser1982Argfs) and c.7913_7917del (p.Phe2638Ter). A total of 5 to 10 mL of peripheral blood was taken for DNA isolation. DNA extraction was performed using the non-enzymatic method [28].

The PCR reaction was performed in an automated thermal cycler (LightCycler^®^ Real-Time PCR 480 System, Roche Life Science, Basel, Switzerland). The 5 µL reaction mixture contained: 1 µL (10 ng) of genomic DNA, 0.125 µL TaqMan Assay 40× (Thermo Fisher Scientific, Waltham, MA, USA) (Table 1) and 2.5 µL of reaction mix (GoTaq^®^Probe qPCR Master Mix 2X, Promega, Madison, WI, USA), supplemented to 5 µL with 1375 µL nuclease-free water. The TaqMan-PCR conditions are described in Table 2.

All mutations were confirmed by direct Sanger sequencing. Sequencing reactions were performed using a BigDye Terminator v3.1 Cycle Sequencing Kit (Thermo Fisher Scientific) according to the manufacturer’s protocol. Sequencing products were analyzed on an ABI Prism 3100 Genetic Analyzer (Thermo Fisher Scientific) [23].

*Controls*

To investigate the association of six *BRCA1/2* alleles, which were identified in the 564 Polish women with DCIS in the current study, including *BRCA1* c.5266dup (p.Gln1756Profs) and c.4035del (p.Glu1346Lysfs) mutations and BRCA2 c.658_659del (p.Val220Ilefs), c.3847_3848del (p.Val1283fs), c.5946del (p.Ser1982Argfs) and c.7913_7917del (p.Phe2638Ter) mutations with the risk of DCIS, we assessed the frequencies of these alleles in the control group of 4702 cancer-free women described above, using TaqMan Assays (Thermo Fisher Scientific, Waltham, MA, USA).

### 2.4. Statistical Analysis

The prevalence of the *BRCA1* and *BRCA2* mutations was estimated in 564 patients with DCIS and 4702 cancer-free women. Odds ratios and their 95% confidence limits for DCIS according to *BRCA1* and *BRCA2* status (positive versus negative) were generated using two-by-two tables. We also estimated odds ratios separately for each of the six variants. Statistical significance (*p*-value) was assessed using a Fisher exact test or Chi-squared test, where appropriate. Mean age of diagnosis of DCIS in *BRCA1* carriers versus in non-carriers, and in *BRCA2* carriers versus non-carriers, were compared using a *t*-test. Median age of diagnosis of DCIS in *BRCA1* carriers versus in non-carriers, and in *BRCA2* carriers versus in non-carriers, were compared using the Mann-Whitney test.

To estimate the all-cause survival (cause of death was not available) of women with DCIS, with and without a mutation, we followed the DCIS patients from the date of diagnosis until date of death or February 2020. Mean follow-up time for *BRCA1/2* carriers versus non-carriers was compared using the *t*-test. Median follow-up time for *BRCA1/2* carriers versus non-carriers was compared using the Mann–Whitney test.

We compared the actuarial survival between mutation carriers and non-carriers with DCIS. We calculated an age-adjusted *p*-value for carriers versus non-carriers using Cox regression analysis. We did not estimate hazard ratio for death for carriers versus non-carriers, because there were no deaths reported among carriers. All statistical analyses were performed using MedCalc Version 22.014.

## 3. Results

We tested 564 women with DCIS for six Polish *BRCA1* founder mutations (c.181T>G, c.4035del, c.5266dup, c.3700_3704del, c.68_69del and c.5251C>T) and for four Polish recurrent mutations of *BRCA2* (c.658_659del, c.3847_3848del, c.5946del and c.7913_7917del). In total, a *BRCA1* or *BRCA2* mutation was detected in 15 of 564 cases of DCIS (2.7%).

Seven patients carried a *BRCA1* mutation (1.24%). Among patients with DCIS and a *BRCA1* pathogenic variant, six (1.06%) carried the most common Polish *BRCA1* mutation, c.5266dup (which constitutes about 60% of all Polish *BRCA1/2* mutations) and one (0.18%) had the c.4035del mutation. Other recurrent alleles of *BRCA1* (c.181T>G, c.3700_3704del, c.68_69del and c.5251C>T) were not detected among the DCIS cases.

Eight women with DCIS (1.42%) carried a *BRCA2* mutation. Among the eight patients with DCIS and a *BRCA2* mutation, each of the four genotyped *BRCA2* variants was detected (c.658_659del, c.3847_3848del, c.5946del and c.7913_7917del).

We then investigated the association of six *BRCA1* and *BRCA2* founder mutations with the risk of DCIS using 4702 cancer-free women as a reference group. A *BRCA1* or *BRCA2* mutation was seen in 15 of 564 cases of DCIS (2.66%) and in 24 of 4702 controls (0.51%; OR = 5.33, CI 95% 2.78–10.22, *p* < 0.0001). A *BRCA1* mutation was present in seven (1.24%) cases and in eighteen (0.47%) controls (OR = 3.27, CI95% 1.36–7.87, *p* = 0.01). A *BRCA2* mutation was present in eight (1.42%) cases versus six (0.13%) controls (OR = 11.26, CI95% 3.89–32.58, *p* < 0.0001) (Table 3).

We analyzed the frequency of *BRCA1/2* mutations by age of DCIS diagnosis. Among the 40 women diagnosed with DCIS before the age of 40, the prevalence of *BRCA1/2* mutations was 5% (2/40). A *BRCA1* or *BRCA2* mutation was observed in 5 of 154 (3.25%) women diagnosed with DCIS before the age of 50, compared to 10 of 410 (2.44%) women with DCIS diagnosed above the age of 49 (*p* = 0.6).

The mean and median age of onset was similar for carriers of the *BRCA1* mutations and non-carriers (53.1 vs. 55.4 years, *p* = 0.6, and 54.0 vs. 55.0 years, *p* = 0.6, respectively). The mean age of diagnosis was 6.6 years lower (48.8 years) for carriers of the *BRCA2* mutations than non-carriers (55.4 years), but the difference did not reach a statistical significance (*p* = 0.1). The median age of diagnosis was 5.5 years lower (49.5 years) for carriers of the *BRCA2* mutations than non-carriers (55.0 years), but again, the difference was not statistically significant (*p* = 0.2) (Table 4).

In 145 of the 564 cases, there was a family history of breast cancer (25.7%). The prevalence of the mutations in these 145 women was 3.5%. Among the fifteen women with DCIS and a mutation, five (33.3%) had a family history of breast or ovarian cancer in a first or second-degree relative.

The all-cause survival data were available for all cases of DCIS. The mean follow-up period was 139 months (median, 156; range, 1–270 months; SD, 62.7), including 110 months (median, 95; range, 9–201 months; SD, 66.6) for carriers versus 140 months (median, 158; range, 1–270 months; SD, 62.5) for non-carriers (*p* = 0.1, *t*-test; *p* = 0.1, Mann–Whitney test). There were no deaths reported in the group of 15 mutation carriers (0%) compared to 18 deaths among the 549 non-carriers (3.3%) within the follow-up time (*p* = 1.0). The 10-year survival rate was 100% for carriers compared to 97% for non-carriers. We did not estimate hazard ratios for carriers versus non-carriers, because there were no deaths reported among carriers.

We followed *BRCA1/2* carriers with DCIS on average for 9 years. Of the fifteen cases with the *BRCA1* or *BRCA2* mutations, eleven were treated with a unilateral mastectomy, one with a lumpectomy and three with a bilateral mastectomy. Five patients received radiotherapy. Three of the fifteen cases with the *BRCA1/2* mutations reported invasive cancer in the ipsilateral or the contralateral breast, diagnosed, on average, 6 years from the date of DCIS diagnosis. Two mutation carriers had DCIS with microinvasion and both developed invasive breast cancer after the diagnosis of DCIS (Table 4). Four of the seven *BRCA1* mutation carriers were diagnosed with ER-negative DCIS. Four of the five patients with *BRCA2* (with available hormone receptor status) were diagnosed with ER-positive DCIS.

## 4. Discussion

The major aim of this study was to establish whether or not *BRCA1/2* mutations confer an increased risk of DCIS in the Polish population, and if DCIS may be a part of hereditary breast and/or ovarian cancer syndrome caused by mutations of *BRCA1/2*. To answer these questions, first we tested 564 women with DCIS for the most common Polish founder mutations of *BRCA1/2* (10 alleles), which account for approximately 80% of all mutations detected in these genes in Poland [26,29,30]. About 3% of 564 Polish patients with DCIS tested positive for six alleles of *BRCA1/2* (four in *BRCA2* and two in *BRCA1*).

To establish the relative risk of DCIS associated with these six alleles, we compared their frequencies in the 564 cases and 4702 cancer-free controls. We observed a higher relative risk of DCIS with a *BRCA2* mutation (OR = 11.3, 95% CI 3.9–32.6, *p* < 0.0001) than a *BRCA1* mutation (OR = 3.3, 95% CI 1.4–7.9, *p* = 0.01). Our findings are consistent with a large study from North America and UK that reported a much stronger association with *BRCA2* than with *BRCA1* for incident DCIS [31]. Although our relative risk estimates are higher than those reported in the study from North America and UK (the odds ratios in the USA/UK study were between four and five for *BRCA2*, and two for *BRCA1*), but the confidence limits in our and the previous study are overlapping. Given the observed DCIS risks, and the lifetime risk of DCIS in women (i.e., approximately 1%), *BRCA1* carriers have only a modest increase in the risk of DCIS (about 2%), and *BRCA2* carriers are at moderate to high lifetime risk of DCIS (above 4%). Therefore, DCIS is a part of hereditary breast and ovarian cancer syndrome caused by *BRCA2* mutations and, to a lesser extent, *BRCA1* mutations.

Approximately 1% of women are diagnosed with DCIS during their lifetime in different populations; the rate of in situ breast cancer from 13 autopsies from 1948 to 2010 was estimated to be as high as 9%. Therefore, most DCIS cases remain clinically insignificant and are not diagnosed during life [32,33]. In addition, the detection rate of DCIS, both among *BRCA1/2* mutation carriers and non-carriers, is highly dependent on the surveillance offered, i.e., mammography, breast ultrasound, MRI and preventive mastectomy [34]. In a recent study of MRI surveillance of healthy *BRCA1* and *BRCA2* carriers, Lubiński et al. detected 33 (1.9%) cases of DCIS and 205 (11.7%) cases of invasive breast cancer in 1756 women undergoing regular MRI surveillance, while among 732 women without MRI surveillance, DCIS was detected in 17 (2.3%) and invasive breast cancer in 79 (10.8%) cases [35]. This shows that, on the one hand, we should keep in mind the possibility of pathogenic mutations in women with DCIS, and on the other hand, in women with already diagnosed *BRCA1/2* mutations, we should not ignore the smallest breast lesions, especially if there are calcifications on mammography, a hypoechoic area on ultrasound or non-mass enhancement on breast MRI scans [36,37].

In the current study, we also wished to estimate the contribution of *BRCA1* and *BRCA2* mutations to DCIS burden in Poland [26,29,38]. A *BRCA1* or *BRCA2* mutation was identified in 15 of 564 Polish cases of DCIS (2.7%), including *BRCA2* mutations (four variants: c.658_659del, c.3847_3848del, c.5946del and c.7913_7917del) seen in eight (1.42%) cases and *BRCA1* mutations (two variants: c.5266dup, c.4035del) observed in seven (1.24%) cases. It is not surprising that the major Polish *BRCA1* mutation (which is present with a frequency of above 0.3% in Poland) was most commonly detected among our DCIS cases (six times; 1.1% of all cases). Other *BRCA1/2* mutations were seen one to three times among Polish women with DCIS.

Previous studies from other countries report different frequencies of *BRCA1/2* mutations among DCIS cases, i.e., depending on ethnic group, the age of diagnosis, the proportion of cases with a family history of breast and/or ovarian cancer. Despite differences in the mutation frequencies in various studies, a greater number of *BRCA2* than *BRCA1* mutations among DCIS cases are consistently found. For example, Petirdis et al. studied 655 British women under the age of 40 with pure DCIS and observed that the *BRCA2* mutations were more common than the pathogenic *BRCA1* variants (3.5% vs. 0.6%) [18]. Evans et al. reported the *BRCA2* mutation rate of 6.28% and the *BRCA1* mutation rate of 2.84% in 311 patients with DCIS [17]. Claus et al. found that 2.4% of 369 women with DCIS had pathogenic variants in *BRCA2* and 0.8% in *BRCA1* [16]. Liu et al. studied 49 Japanese women with DCIS, and found that 18.4% of women tested positive for *BRCA1/2* mutations, 16.3% for *BRCA2* and 2% for *BRCA1* [21]. Hall et al. analyzed a large group of 7295 patients with ductal and lobular carcinoma in situ and found that 5.6% of women had *BRCA1/2* mutations [39]. On the other hand, the Breast Cancer Association Consortium studied 4187 women with DCIS and identified a mutation of *BRCA1* in 0.24% and of *BRCA2* in 0.66% of cases [40]. In our study, *BRCA2* mutations were marginally more common than *BRCA1* mutations (*BRCA2*—1.4% vs. *BRCA1*—1.2%), and we saw an overall 2.7% prevalence of the mutated *BRCA1/2* alleles in Polish women with DCIS. The mutation spectrum seen among our patients may be explained by the fact that *BRCA1* mutations are much more common than *BRCA2* mutations in Poland, in particular, due to the presence of the c.5266dupC founder allele of *BRCA1*, which constitutes about 44–60% of all *BRCA1/2* mutations seen in Poland and is detected with a frequency of 0.17–0.3% in the Polish population [41,42,43,44,45].

In this study, we showed a clinical characteristic of DCIS cases among *BRCA1/2* carriers that may be useful for future meta-analyses. Most Polish women with DCIS were diagnosed with high-grade tumors. Among Polish cases with DCIS and *BRCA2* pathogenic variants, most had ER-positive and PR-positive tumors. Contrastingly, most carriers of *BRCA1* mutations and DCIS were diagnosed with ER-negative and PR-negative tumors. This is in line with previous findings. Liu et al. showed ER-positive status, PR-positive status and a higher grade are independent significant predictors for a *BRCA2* mutation [21]. Petirdis et al. showed that *BRCA2* mutations were associated with ER-positive DCIS, while *BRCA1* mutations were predominantly associated with ER negative DCIS [18].

We also analyzed the survival of DCIS patients. The mean follow-up period was 139 months and the 10-year mortality was 3% among 564 Polish DCIS patients. According to Elshof et al. at 10 years, the cumulative breast cancer mortality among DCIS patients is 2.3% for women <50 years and 1.4% for those >50 years at diagnosis [46]. Interestingly, we saw no deaths among the 15 Polish *BRCA1/2* mutation carriers within the follow-up period. This contrasts with the 23% mortality rate seen at 15 years among about 500 Polish women with *BRCA1* mutations [27]. Although the group of *BRCA1/2* carriers with DCIS was small, our study suggests that *BRCA1/2* mutations predispose patients to a good DCIS prognosis. If the scenario is true (that *BRCA1/2* mutations are associated with good DCIS prognosis), it is possible that there are genetic and/or environmental modifiers predisposing DCIS in *BRCA1/2* mutation carriers, which is associated with better survival [47].

This is the first analysis of the contribution of *BRCA1/2* mutations to this disease in Polish women. Our study was large, as we conducted a case-control (association) analysis that included 564 cases of DCIS and 4702 controls, all from Poland. We examined survival among patients with DCIS including those with a *BRCA1* or *BRCA2* mutation. Our study was characterized by a long follow-up period and reliable survival data. This study also benefited from the genetic homogeneity of the Polish population and the presence of common founder alleles of *BRCA1* and *BRCA2* in Poland. We tested for the ten most common Polish founder alleles of *BRCA1* and *BRCA2*, and the sensitivity of our mutation testing is estimated to be 80%. *BRCA1* and *BRCA2* sequencing is needed to describe the full spectrum of mutations in the two genes in Polish women with DCIS.

The aim of our control group was to calculate with accuracy the frequency of *BRCA1/2* variants in the underlying Polish population. We used a large cohort of cancer-free women to maximize the number of controls, as the studied variants are uncommon in the general population (combined frequency of 0.5%). The median age of our case (55.0 years, SD 10.8) and control (54.0 years, SD 11.5) participants was similar. Moreover, the variant frequency was similar in younger and older controls. Case and control participants were all Polish women.

*BRCA1* and *BRCA2* genetic testing is used in routine practice in Poland and elsewhere. Poland has a genetically homogenous population, and that is expressed in the presence of Polish *BRCA1/2* founder mutations. Our clinical genetic testing, in particular, benefits from the presence of five of the most common mutations of *BRCA1* (c.181T>G, c.4035del, c.5266dup, c.3700_3704del, c.68_69del and c.5251C>T), which constitute about 77% of all *BRCA1/2* mutations detected in Polish women with breast cancer [26]. According to Polish guidelines, a panel of these five founder mutations of *BRCA1* is used as an initial screening tool for all patients with breast cancer and/or ovarian cancer, and healthy individuals with a family history of at least breast and/or ovarian cancer. Full sequencing of *BRCA1/2* using NGS is performed in women with breast cancer diagnosed below the age of 45 regardless of family history, in all women with triple negative breast cancer, in all women with a family history of breast and/or ovarian cancer, all men with breast cancer and all women with ovarian cancer [2,48]. We use standard management for unaffected *BRCA1/2* mutation carriers, including breast MRI, preventive mastectomy and oophorectomy and, individualized treatment for breast cancer patients with a *BRCA1/2* mutation [35].

In Poland, women with preinvasive breast cancer (in contract to those with invasive cancer) are not commonly referred to an outpatient genetic clinic [49]. Therefore, on the basis of our study, we encourage clinicians to refer patients with DCIS for clinical genetic testing.

## 5. Conclusions

In conclusion, DCIS is part of the hereditary breast and ovarian cancer syndrome caused by *BRCA1/2* mutations. Therefore, women with DCIS should receive genetic counseling and testing for *BRCA1/2* mutations. We consider DCIS patients with a positive test result to be candidates for *BRCA1/2*-specific surveillance and possibly mastectomy or bilateral mastectomy. It is likely that *BRCA1/2* mutations predispose individuals to a good DCIS prognosis, but further studies are needed in this regard. It is also important to monitor patients with DCIS and microinvasion, as these patient may face a relatively high risk of developing invasive cancer.

## Figures and Tables

**Table 1:** TaqMan Assays used to detect BRCA1/2 Polish recurrent mutations.

AssayID	HGVS	dbSNP
Pre-designed Assays		
C_164414391_20	BRCA1 c.5266dup (p.Gln1756fs)	rs80357906
C_153129912_20	BRCA1 c.4035del (p.Glu1346fs)	rs80357711
C_153129972_20	BRCA1 c.3700_3704del (p.Val1234fs)	rs80357609
C__32953549_10	BRCA1 c.5251C>T (p.Arg1751Ter)	rs80357123
C_154784555_20	BRCA2 c.658_659del (p.Val220fs)	rs80359604
C_154784896_20	BRCA2 c.3847_3848del (p.Val1283fs)	rs80359405
C_154785160_20	BRCA2 c.5946del (p.Ser1982fs)	rs80359550
C_154785471_20	BRCA2 c.7913_7917del (p.Ala2637_Phe2638insTer)	rs80359686
Custom-design Assays		
Not available	BRCA1 c.181T>G (p.Cys61Gly)	rs28897672
Not available	BRCA1 c.68_69del (p.Glu23fs)	rs80357914

**Table 2:** PCR conditions used in all TaqMan-PCR reactions.

Program Name	Target (°C)	Acquisition Mode	Hold (hh:mm:ss)	Ramp Rate (°C/s)	Acquisition (per °C)	Cycles	Analysis Mode
Pre-incubation	95	None	00:10:00	4.8		1	None
Amplification	95	None	00:00:10	4.8		45	Quantification
	60	None	00:00:30	2.5			
	72	Single	00:00:01	4.8			
Cooling	40	None	00:00:30	2.5		1	None
Melt	60	None	00:00:01	4.8		1	Melting curves
	61	Continuous		0.06	5		
Cooling	40	None	00:00:30	2.5		1	None

**Table 3:** Prevalence of Polish *BRCA1* and *BRCA2* recurrent mutations in 564 women with DCIS and in 4702 cancer-free controls, with corresponding odds ratios.

	DCIS N = 564	Controls N = 4702		
Mutation	Positive (%)	Positive (%)	OR (CI 95%) *	*p*-Value
***BRCA1***				
c.5266dup	6 (1.06%)	16 (0.34%)	3.15 (1.23–8.08)	0.03
c.4035del	1 (0.18%)	2 (0.04%)	4.17 (0.38–46.13)	0.74
Any *BRCA1*	7 (1.24%)	18 (0.38%)	3.27 (1.36–7.87)	0.01
***BRCA2***				
c.7913_7917del	3 (0.53%)	1 (0.02%)	25.14 (2.61–242.22)	0.0008
c.658_659del	2 (0.35%)	3 (0.06%)	5.57 (0.93–33.45)	0.16
c.3847_3848del	2 (0.35%)	1 (0.02%)	16.73 (1.51–184.90)	0.03
c.5946del	1 (0.18%)	1 (0.02%)	8.35 (0.52–133.77)	0.51
Any *BRCA2*	8 (1.42%)	6 (0.13%)	11.26 (3.89–32.58)	<0.0001
**Any *BRCA1/2***	15 (2.66%)	24 (0.51%)	5.33 (2.78–10.22)	<0.0001

* Odds ratios and *p* values were calculated using cancer-free Polish women as a reference group.

**Table 4:** Clinical characteristics of women with DCIS and a *BRCA1* or *BRCA2* mutation.

No.	Gene	Mutation	Age	Follow-Up, Months	Pathology	ER	PR	HER2	Two-Sided Adnexectomy	Treatment	Breast Cancer After DCIS, Months
1	*BRCA1*	c.5266dup	46	71	DCIS G3	–	–	–	yes	bilateral mastectomy	no
2			52	111	DCIS G3	+	+	n *	yes	mastectomy	no
3			54	201	DCIS G3	+	+	n *	yes	bilateral mastectomy, radiotherapy	no
4			54	83	DCIS G3	–	–	–	yes	bilateral mastectomy	contalateral 61 months
5			56	95	DCIS G2	+	+	n *	yes	mastectomy, radiotherapy	no
6			58	78	DCIS G2	–	–	–	yes	mastectomy	no
7		c.4035del	52	155	DCIS G3 microivasion	–	–	+	yes	mastectomy, radiotherapy	contalateral 79 months
8	*BRCA2*	c.658_659del	29	79	DCIS G3 microivasion	+	+	+	no (none)	mastectomy, radiotherapy	ipsilateral 84 months
9			48	17	DCIS G1	n *	n *	n *	no (none)	mastectomy	no
10		c.7913_7917del	36	22	DCIS G3	–	–	–	no (none)	mastectomy	no
11			51	178	DCIS G3	+	+	n *	yes	mastectomy	no
12			66	195	DCIS Gx	m *	m *	m *	m *	mastectomy	no
13		c.3847_3848del	41	186	DCIS G2	+	+	n *	yes	mastectomy	no
14			64	9	DCIS G2/3	m *	m *	m *	no (none)	lumpectomy, radiotherapy	no
15		c.5946del	55	166	DCIS G3	+	+	–	no (none)	mastectomy	no

* m—missing data; n—not tested; Gx—grade unknown.

## Data Availability

The main research data supporting the results of this study are included in Table 3 and Table 4. Other data could be shared from the authors upon request.
